# Multispectral tissue characterization for intestinal anastomosis optimization

**DOI:** 10.1117/1.JBO.20.10.106001

**Published:** 2015-10-06

**Authors:** Jaepyeong Cha, Azad Shademan, Hanh N. D. Le, Ryan Decker, Peter C. W. Kim, Jin U. Kang, Axel Krieger

**Affiliations:** a Johns Hopkins University, Department of Electrical and Computer Engineering, 3400 North Charles Street, Baltimore, Maryland 21218, United States; b Sheikh Zayed Institute for Pediatric Surgical Innovation, Children’s National Health System, 111 Michigan Avenue, Washington, DC 20010, United States

**Keywords:** multispectral imaging, cross-polarization, tissue classification, intestinal anastomosis

## Abstract

Intestinal anastomosis is a surgical procedure that restores bowel continuity after surgical resection to treat intestinal malignancy, inflammation, or obstruction. Despite the routine nature of intestinal anastomosis procedures, the rate of complications is high. Standard visual inspection cannot distinguish the tissue subsurface and small changes in spectral characteristics of the tissue, so existing tissue anastomosis techniques that rely on human vision to guide suturing could lead to problems such as bleeding and leakage from suturing sites. We present a proof-of-concept study using a portable multispectral imaging (MSI) platform for tissue characterization and preoperative surgical planning in intestinal anastomosis. The platform is composed of a fiber ring light-guided MSI system coupled with polarizers and image analysis software. The system is tested on *ex vivo* porcine intestine tissue, and we demonstrate the feasibility of identifying optimal regions for suture placement.

## 1 Introduction

Over a million anastomoses are performed in the United States each year for visceral indication alone (gastrointestinal, urological, and gynecological surgeries).^1^ To date, intestinal anastomosis surgeries are performed either openly or through minimally invasive techniques using sutures or mechanical staplers.^2^^–^^5^ Despite the routine nature of intestinal anastomosis procedures, the rate of complications such as anastomotic leakage and strictures is between 3% and 19% and remains unchanged despite the introduction of newer techniques and technologies.^6^^,^^7^ These complications undermine the clinical outcomes and often require repeated surgery, leading to a significant increase in treatment cost, morbidity, and mortality.^8^ Generally, suturing techniques such as suture placements are guided by the surgeon’s visual perception. Although there have been remarkable advances in surgical imaging systems^9^^,^^10^ and contrast-enhancing methods^11^ for improving surgical vision,^12^ it is desirable to have optical imaging tools to guide and improve the surgeon’s intraoperative decisions and facilitate anastomosis with a clearer target-to-background tissue contrast to improve surgical outcomes.

Multispectral imaging (MSI) is an advanced imaging technique to capture scene information at different spectral wavelengths, which has been used to spatially and spectrally classify similar materials according to their distinguished signatures.^13^ Multispectral images show structural properties that may be invisible using a single wavelength and can also reveal subsurface features at longer wavelengths, such as near-infrared light. Various biomedical applications,^14^ such as cancer detection^15^ and blood oxygen saturation observations in skin,^16^ have been reported by employing this technique. On the other hand, polarization-sensitive imaging (PSI) uses the scattering and polarization properties of light propagating in the tissue.^17^ When incident light strikes the tissue surface, a portion of the light is reflected as specular reflection, while another portion propagates through the tissue. The light propagating through the tissue is depolarized. However, the Fresnel reflection from the surface retains the original polarization state. By considering the fact that the difference in the polarization states depends on the light penetration depth, polarization control techniques are often used for depth-selective measurements.^18^ In addition, cross-polarization imaging methods can be used to eliminate specular reflection from the tissue surface, allowing clear identification of subsurface structures, which is often required for surgical procedures.^19^^,^^20^

In this study, we demonstrate an MSI platform that offers a guide to surgeons for optimum suture placement in bowel anastomosis. This platform provides a novel combination of an MSI system with PSI for analyzing spatial and spectral data acquired from tissues at all points across the measured imaging area. To our knowledge, MSI for displaying subsurface tissue information beyond the human visual spectrum to guide and optimize suture placements has not been applied to date. We used *ex vivo* porcine small intestinal tissue to evaluate and characterize the system performance, as the morphology and size are similar to human small intestine. Although *ex vivo* tissue does not possess blood flow, tensile strength, or tissue perfusion similar to those of intact live tissue, their anatomical tissue characteristics such as blood vessels, thickness, and tissue types remain unchanged. Our data on the spatial and spectral characteristics of the tissues, which were obtained from MSI images, were further processed to identify blood vessels, differentiate between thin and thick tissue areas, and segment different tissue regions. Blood vessel avoidance is clinically important to limit bleeding and retain blood supply to the suture site for healing.^21^ Thicker tissue areas have higher mechanical and suture retention strength and are more suitable for suture placement. Predicting the mechanical strength of tissue is highly relevant in robot-assisted surgical procedures with limited haptic feedback as well as in pediatric surgeries where anastomosis is performed in often paper-thin tissue and long tissue gaps exist between ends, requiring large forces to approximate and secure the ends.^22^ Tissue thickness also influences the ideal suture bite size, which is typically recommended as 1.5 times tissue thickness.^23^ Tissue classification is important to identify the cut line and the area of the tissue within the surgical field that needs to be sutured. The segmentation and identification resulted in a numerical topographic suture map corresponding to desirable suture locations, which could assist surgeons in suturing.

## 2 Materials and Methods

**Fig. 1:**
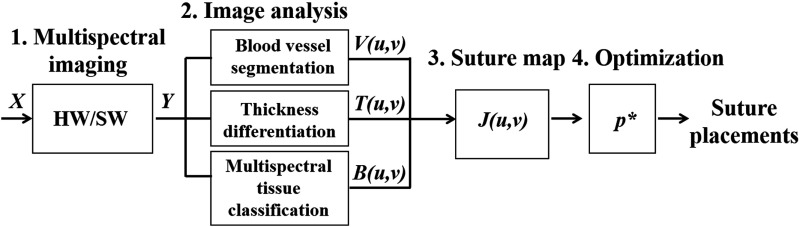
System block diagram to create recommendations for optimal suture placements.

Our method consisted of four main steps (Fig. 1). First, for data acquisition, a portable MSI system (hardware/software) acquired raw data (X) and output data (Y) of multiple single-band images for image analysis. Second, three submaps were created by blood vessel segmentation, thickness differentiation, and multispectral tissue classification. Specifically, in the blood vessel map, for example, pixels which confidently belong to a blood vessel are assigned a value of 0 and everything else a value of 1. A two-dimensional (2-D) Gaussian smoothing is used to generate values between 0 and 1 to create a vessel-possibility map around the confidently segmented blood vessels. Thick tissues that could be sutured well were also assigned a value of 1 and thin tissues were assigned a value of 0, and 2-D Gaussian smoothing filtered values were assigned to tissue regions between confident-thin (0) and confident-thick (1) areas. Additionally, morphological image processing of the multispectral tissue classification output identifies the bowel cut section, from which a submap for bite depth can be created. Third, given the parameters from the image analysis, a suture map (J) was generated by combining the submaps using an elementwise matrix multiplication operator, where high-intensity pixels correspond to desirable suture locations. Fourth, an optimization technique identified local peaks in the suture map as candidates for desired suture locations. Equidistant suture placements are chosen from the candidates based on the recommended intersuture distance of 1.5 times the tissue thickness to help surgeons prioritize and identify the areas that are suitable for suture placements.

### 2.1 Implementation of the Multispectral Imaging System

**Fig. 2:**
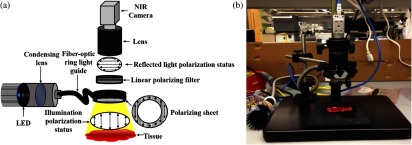
(a) Schematic of the multispectral imaging (MSI) system and (b) photo of system implementation.

A schematic of the MSI platform is presented in Fig. 2. A predetermined narrowband high-power light-emitting diode (LED) light source (SR-02, Quadica Developments Inc., Ontario, Canada) was used with a fiber-optic ring light guide and a condensing lens to generate three uniform illumination lights (center wavelengths 470, 600, and 770 nm) in series. These wavelengths in the visible spectrum were selected to demonstrate hemoglobin absorption and to examine the effects of wavelengths on penetration depth.^24^ Since the LED light was unpolarized and the use of nonpolarization-maintaining fibers randomized the polarization state of the light,^25^ we applied a polarizing sheet onto the distal end of the ring light guide to create linearly polarized illumination. Reflected light from the tissue passes back through the empty space of a ring light guide, a rotating linear polarizer filter (46 mm, Prinz Optics GmbH, Stromberg, Germany), a macrolens (Fujinon HF 12.5 SA-1, Phoenix Imaging Ltd., Michigan), and finally reaches a near-infrared camera (acA2000-50gmNIR, Basler, Pennsylvania). By adjusting the angle of the linear polarizer attached to the camera, we can effectively control the amount of polarization effects. To reduce specular reflections from the tissue surfaces, two linear polarizers were set orthogonal to each other. Three different spectral images were acquired at 6 fps, with the image size of 1280×1080  pixels. LED-based MSI has an advantage over hyperspectral imaging in that it enables high-speed image acquisition and data processing, which could be potentially useful for real-time guidance. Both LED control and image acquisition were programmed using a custom C# script (Visual Studio 2010, Microsoft). The acquired images were cropped to obtain the tissue region of interest (735×637  pixels) for image processing. The 470-nm band images were selected for blood vessel segmentation.^26^ In addition, all three spectral band images were combined to form composite images that were used for the multispectral analysis.

### 2.2 Animal Tissue Preparation

Fresh porcine small bowels were obtained from a local abattoir and dissected into segments of 20 to 30 cm long. The sample was moistened with physiological saline and preserved at 4°C for up to 30 h from the time of slaughter until imaging. Before imaging, different segments of the small and large bowels were dissected into 5-cm-long specimens. During the measurements, the remaining samples were preserved in saline in sealed sterile containers for hydration maintenance for up to 30 min.

### 2.3 Image Analysis and Suture Map

In this study, three tissue maps of blood vessel segmentation (V), thickness differentiation (T), and bite depth based on multispectral tissue classification (B) were produced.

– Blood vessel segmentation: vasculature structure is identified by applying a 2-D filter^27^ on the single-band cross-polarized 470-nm channel. The filter identifies and segments vessels by examining the Hessian of the image and measuring the eigenvalues of the Hessian.^27^ The resulting blood vessel map is negated to assign the value of 0 to blood vessels. We further smoothed the vessel map with a 2-D Gaussian to avoid neighboring pixels.
– Tissue thickness differentiation: the images obtained at 470, 600, and 770 nm were evaluated using a supervised spectral angle mapper (SAM) method.^28^^,^^29^ The SAM technique characterizes the spectral similarity between individual pixels of a sample and *a priori* reference by computing the angle of difference between their spectral vectors. We chose as a reference an averaged spectrum of five arbitrary, nonoverlapping tissue regions within a similar tissue thickness. The outcome of SAM was an abundance map that resembled the original image, with spectral signature information of each tissue type. In our application, we predefined the endmember references to be the double-layered (nonincised) and single-layered (incised) tissue areas as the spectral library for SAM method. The SAM extracted features were used to confirm the thicker tissue area within the surgical suture site. A similar smooth kernel, as explained in blood vessel segmentation, is applied to SAM’s extracted thicker tissue endmember to indicate local maxima for the final suture map convolution.
– Bite depth from multispectral tissue classification: the acquired multispectral images were analyzed using the image analysis software MultiSpec, a freeware multispectral image data analysis system.^30^ By creating a composite image from multispectral images, four different regions (background; inner lumen, i.e., mucosa and submicosa; outer lumen, i.e., serosa; and mesentery) were manually defined by designating training fields, and the discriminant analysis was performed to classify the corresponding regions. The inner and outer lumens were then used to extract the boundary of the cut section, where sutures should be placed. The distance of suture placement from the cut section is also referred to as the “bite depth.” Normally, surgeons choose a bite depth of 1.5 times the thickness of the lumen.^23^ We create a bite depth map which approximates this empirical rule.

The combination of the above three mentioned tissue maps resulted in the suture map. The intensity levels of the suture map, J(u,v), range from 0 to 1, where 0 denotes a location that should absolutely be avoided for suture and 1 denotes a location that could be used for suture with minimal complications. The fusion operator used to combine these different matrix maps into a single suture map is the elementwise matrix multiplication:

J=V⊗T⊗B, (1)

where V is the blood vessel segmentation map, T is the thickness differentiation map, B is the map obtained from processing the multispectral tissue classification, and ⊗ is the elementwise matrix multiplication operator, that is, J(u,v)=V(u,v)×T(u,v)×B(u,v), where u and v are the horizontal and vertical pixel indices.

The optimal suture points could be calculated automatically by solving the following optimization problem locally:

$$p^{*}=\underset{p=[u,v]}{\arg\;\max}\;J(u,v),$$ (2)

where $p^{*}=[u^{*},v^{*}]$ is a local maximum of the suture map J.

This optimization problem is not convex and does not have a global maximum. Local maxima were extracted as candidates for suture placements. A computationally efficient method to solve this nonlinear optimization problem approximately is to first eliminate all the pixels that are smaller than a threshold. The remaining pixel values are then compared with their eight neighbors. If a value is larger than all eight neighbors, then it is kept as a local maximum. This method will find most of the local maxima in the suture map image quickly. Using an equidistance consistency constraint, the candidate list of placements can be refined to include only equidistant suture placement recommendations. The final list of recommendations along with a colormap visualization of the suture map J is provided to the surgeon to make informed decisions on avoiding vessels, choosing thick tissue to retain stronger forces, and be at an accepted distance from the lumen cut section.

## 3 Results

### 3.1 Multispectral Imaging

**Fig. 3:**
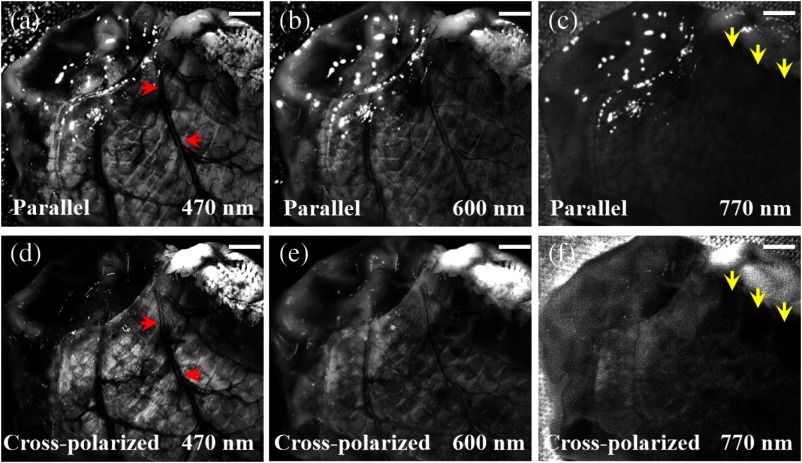
Different spectral band images of a tissue sample with (a)–(c) and without cross-polarization, (d)–(f) demonstrating surface reflection removal. Arrows indicate features of blood vessels in red color (a) and (d) and the revealed subsurface features in yellow color. (c) and (f) White scale bars: 2 mm.

Figure 3 shows a porcine intestinal tissue imaging result at three different wavelengths, where the superficial features, including the blood vessels, are accentuated at 470 nm (red arrows). At 770 nm, light penetrates deeper within the tissue, revealing subsurface features [yellow arrows in Figs. 3(c) and 3(f)]. This figure also demonstrates that the cross-polarization scheme can successfully eliminate surface reflections such as glare from the tissue. Note that as the illumination wavelength band increases from blue to red and near-infrared, the image contrast decreases owing to the increased scattering and mean free path at longer wavelengths.

### 3.2 Blood Vessel Map

**Fig. 4:**
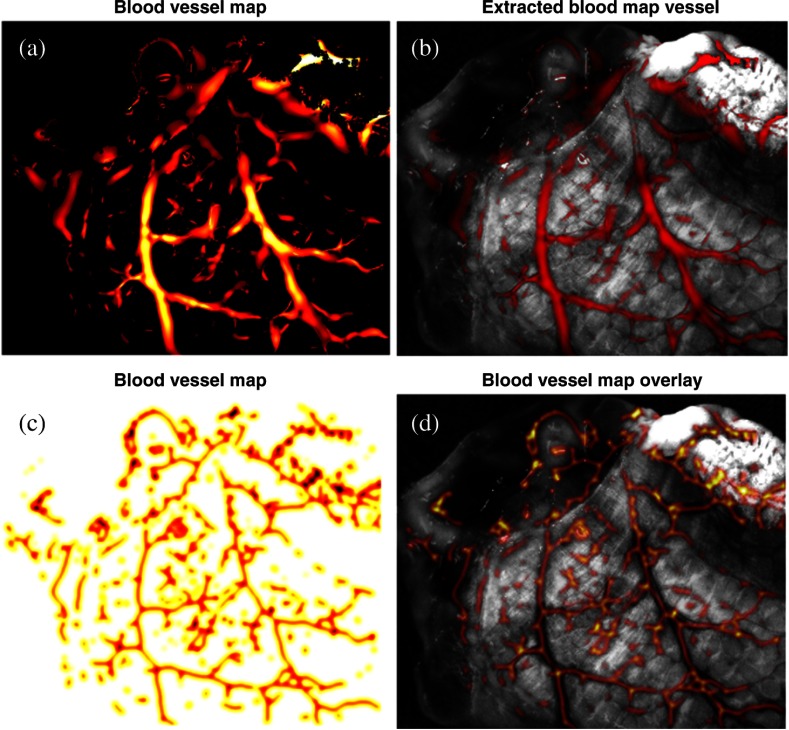
(a) Blood vessel segmentation using Frangi 2-D filter.^24^ (b) Blood vessel segmentation result (red) image overlay on the single-band image at 470 nm. (c) Blood vessel map V(u,v) created by Gaussian filter smoothing of the output of the Frangi 2-D filter. (d) Image overlay of inverted vessel map (inverted for better visualization) on the single-band reflectance image of the intestine.

Figure 4(a) shows the result of the 2-D Frangi filter^27^ on the single-band cross-polarized 470-nm channel, which identifies the vasculature structure using a colormap. The values range from 0 to 1, where larger values correspond to a more confident identification of a blood vessel. Figure 4(b) overlays the result in red on the input image for visualization and comparison purposes. The blood vessel map in Fig. 4(c) is extracted from the vasculature structure by negating and Gaussian filter smoothing. The dark areas identify blood vessels that should be avoided to prevent stricture. Blood vessel avoidance is achieved by elementwise multiplication of the blood vessel map to other maps. Figure 4(d) visualizes the blood vessel map on the input 470-nm band image.

### 3.3 Thickness Map

**Fig. 5:**
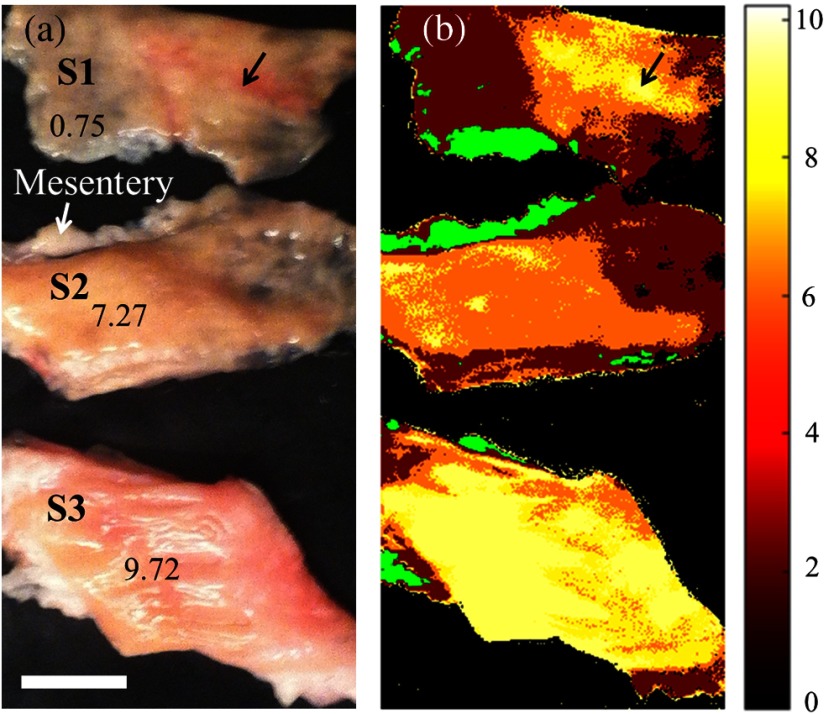
(a) Digital photograph of three bovine colon tissues with different specified thicknesses (units in mm). (b) Thickness differentiation using the SAM method, with a thickness-corresponding colormap. The mesentery is indicated as a different tissue in green color. Black arrows: thicker tissue areas; white scale bar: 10 mm; and colormap unit: mm.

Thickness differentiation was performed using SAM.^28^^,^^29^ A pilot study for thickness measurement, as demonstrated in Fig. 5, involved the use of three controlled bovine colon samples with layer heights of 0.75 mm (S1), 7.27 mm (S2), and 9.72 mm (S3). The mesenteries attached to the intestine were considered as separated tissues, which were extracted before the thickness analysis.

Figure 5(b) shows the analyzed tissue thickness indicated by a heat colormap to represent thickness ranging from 0 to 10 mm. The result was mostly consistent with the measured physical dimensions of each sample, as shown in Fig. 5(a). In addition, it also indicates the effect of tissue types on thickness analysis, especially in the situation of a thin nonhomogenous sample such as S1, where blood vessel at similar height as S2 is classified as having the same thickness as the sample S3 (Fig. 5, black arrows).

**Fig. 6:**
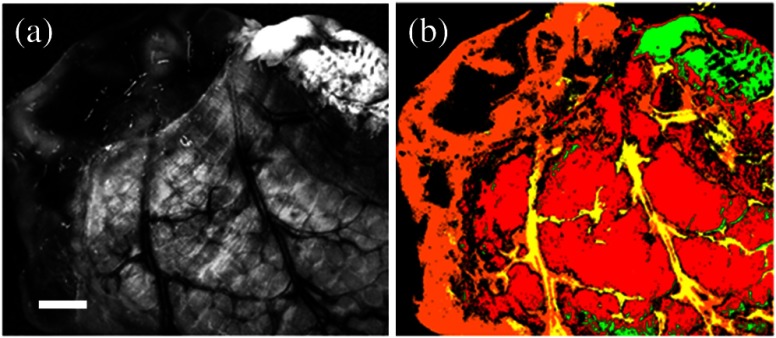
(a) Representative single-band reflectance image at 470 nm. (b) Thickness differentiation using the SAM method, with a thickness-corresponding colormap. The red color shows the thicker layer, and the orange color shows the thinner layer. Tissue classification of the mesentery (green) and blood vessels (yellow) was performed prior to the thickness analysis. White scale bar: 2 mm.

Similarly, we applied the same SAM method to the multispectral images of the same porcine intestinal tissue in order to extract thick tissue area, as it is highly influenced on applied suture tension and bite size. Bowel wall thickness increases with age from 0.5 mm for infants to 2.0 mm for adults based on ultrasounds^31^ and is generally thicker than 0.9 mm in healthy pediatric and adult subjects.^32^ Thus, we are considering a lower limit of wall thickness for suturing to be 1 mm. We prepared our bowel tissue sample with a scalpel to contain a thinned-out section with thickness <1  mm on the left (Fig. 6, orange, corresponding to a 0 for the suture map calculations) and a thicker section of about 2 mm thickness on the right (Fig. 6, red, corresponding to a 1 for the suture map calculations). Figure 6 shows the tissue thickness analysis using the supervised SAM method with predefined endmember references of thicker tissue section at the double-layered intestinal region (in red color) and thinner tissue section at the single-layered intestinal section (in orange color). The single-layered region indicates the incised section, while the double-layered one indicates a nonincised region. Prior to the SAM analysis, the image is analyzed to segment features such as the mesentery (in green) or blood vessels (in yellow).

**Fig. 7:**
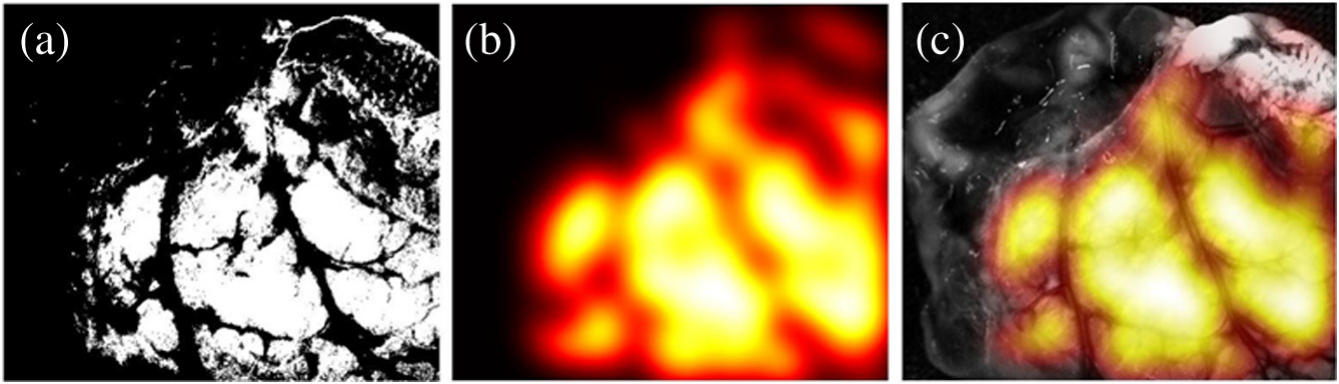
(a) Thickness binary map as evaluated by the SAM algorithm. (b) Smoothed thickness map T(u,v). Larger values (brighter) denote areas with thicker tissue, which are better suited for suture placement. (c) Overlay of thickness map over a single-band image for better visualization.

We further implemented a smooth Gaussian kernel convolution on this nonincised region. The smoothing kernel is used on the thickness binary map to signify the necessary thick tissue density for the convolution of B(u,v) and V(u,v) maps (Fig. 7).

### 3.4 Multispectral Tissue Classification and Bite Depth Map

**Fig. 8:**
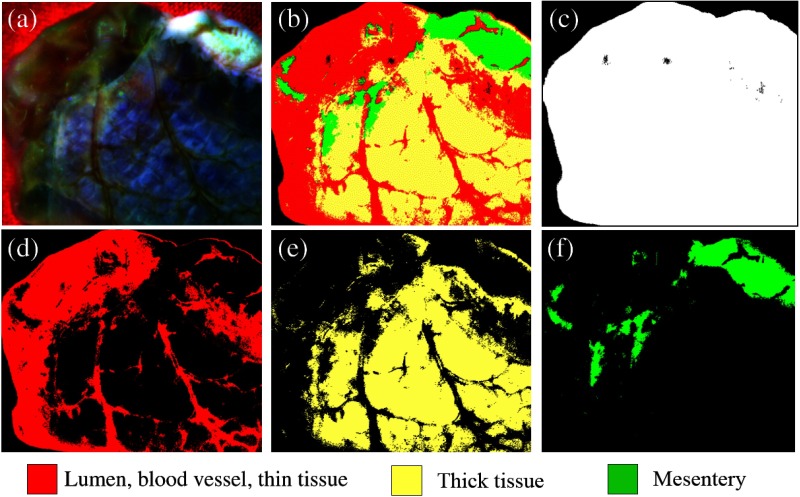
Multispectral tissue classification: (a) composite image created from three spectral band images; (b) classified image using the supervised classification algorithm; (c) background color-matching image (black); (d) image showing the vulnerable tissue (lumen, blood vessels, and thin tissue regions) (red); (e) image showing the thick tissue regions (yellow); and (f) image showing the mesentery (green).

**Fig. 9:**
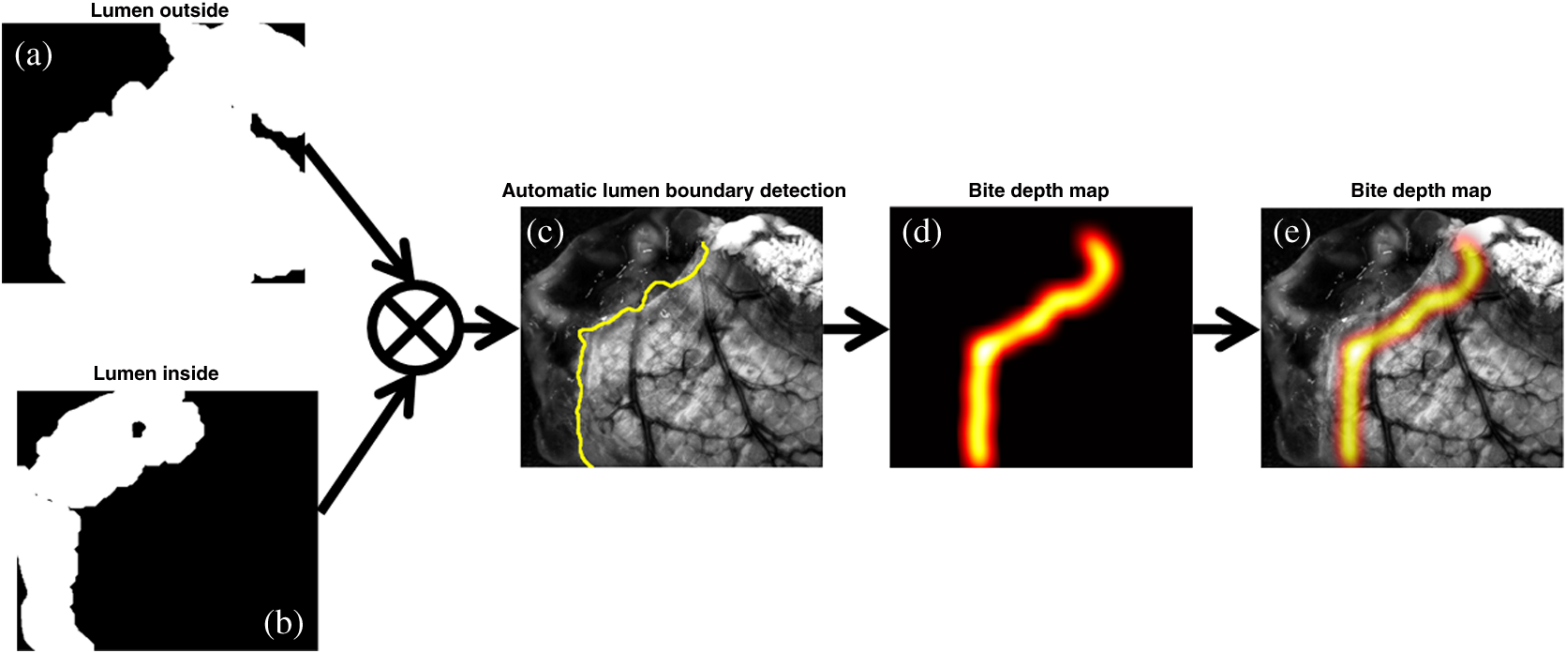
Multispectral image analysis facilitates segmentation of (a) outer layer of the lumen (serosa) and (b) inner layers of the lumen (mucosa and submucosa layers). (c) The cut edge is automatically extracted by pixelwise multiplication of a dilated map of outer and inner layers of the lumen. (d) A bite depth map B(u,v) is generated by translating and smoothing the cut edge by 1.5 times the thickness of the tissue. (e) Overlay of the bite depth map on a single-band image for better visualization.

The multispectral tissue analysis results using the MultiSpec program are shown in Fig. 8. The composite image [Fig. 8(a)] of the porcine intestinal tissue was created from three spectral band images of the background, lumen (mucosa and submucosa layers), blood vessels, thick tissue outer layer of serosa, and the mesentery. Those four regions were segmented in different colors [Fig. 8(b)]. The foreground mask is shown in Fig. 8(c). The lumen, blood vessel, and thin tissue areas of serosa and mesentery are shown in red [Fig. 8(d)] and yellow [Fig. 8(e)], respectively, whereas the mesentery is depicted in green [Fig. 8(f)]. Although a small tissue portion including the blood vessels was indicated in red, the program successfully segmented the inside and outside tissue areas and the mesentery. The blood vessels are accurately accounted for in the final map, using the specific blood vessel segmentation results (Sec. 3.2). We can observe that lumen, blood vessel, thin and thick tissue regions of the intestine, and mesentery are successfully segmented by the algorithm after repetition of training sets for several tissue types as supervised by the user. The inside (mucosa and submucosa) and outside (serosa) of the lumen can be used to extract the edge between the two regions, which outlines the cut line [Figs. 9(a)–9(c)]. The cut line is used as a reference to place sutures at a certain distance for better healing. To extract the edge regions, we used the standard binary image processing methods of dilation and erosion in MATLAB (R2015a, Mathworks Inc., Massachusetts). This line [Fig. 9(c)] was used to determine the bite size distance, given the tissue type and size. In surgery, the rule of thumb for the general suture technique is to calculate the suture placement distance from the tissue cut end (suture width) as 1.5 times the tissue thickness, τ.^23^ The computed bite size distance was convolved with a smooth Gaussian to account for uncertainty in the bite size distance computation. The standard deviation σ of the Gaussian filter is chosen to give more weight to points that are at 1.5τ (mm) distance but are not closer than δ mm from the cut edge. For 99.7% of the filtered values to be in this range (3σ-rule), σ=(1.5τ−δ)/3. The value of δ depends on the suture size but usually should not be smaller than 0.5 mm. With a tissue thickness of 1 mm for this sample, σ=0.33  mm. The resulting map, B(u,v), is depicted in Figs. 9(d) and 9(e).

### 3.5 Suture Map and Suture Placement Recommendations

**Fig. 10:**
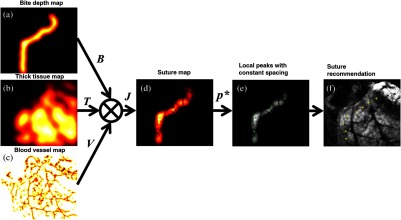
Suture map and suture placement recommendations: (a) bite-depth map B(u,v); (b) thick tissue map T(u,v); (c) blood vessel map V(u,v); (d) combined map J(u,v); (e) selection of local peaks with equal-space consistency constraint; and (f) an overlay image of the recommended suture locations.

**Fig. 11:**
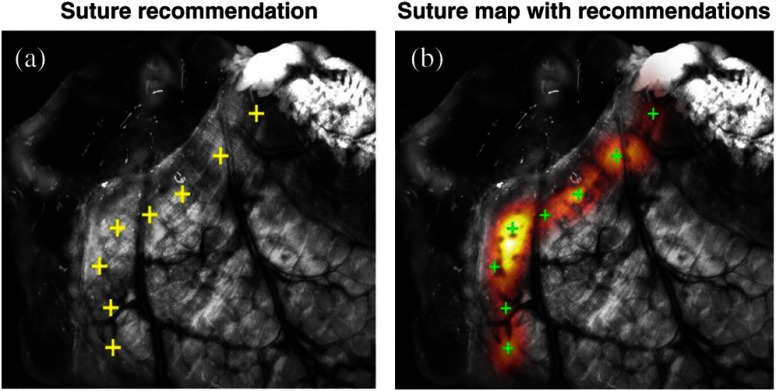
(a) Magnified view of the suture placement recommendations and (b) colormap overlay of the suture map provided to the surgeon to overrule recommendations and choose other acceptable regions.

Finally, a suture map including the anatomical and geometrical information was generated using the smooth gradients from the individual image analyses (Fig. 10). The bite depth, tissue thickness, and blood vessel maps [Figs. 10(a)–10(c)] are combined using a multiplier operator to create the suture map, as shown in Fig. 10(d). Suture placement recommendations are the local maxima from the suture map and are depicted in Fig. 10(e) with the overlay image shown in Fig. 10(f). At the end, the surgeon is provided both the recommendations as well as a colormap overlay of the acceptable suture locations on the image, so that they can decide if they want to overrule the recommendation of the software (Fig. 11).

## 4 Discussion

Providing surgeons with subsurface tissue information beyond standard surface shapes and patterns obtained using current surgical imaging techniques may improve the surgeon’s decision making and lead to better surgeries and reduced complication rates. Toward this goal, we developed and evaluated an MSI platform for intestinal anastomosis. We showed that the system successfully determines blood vessel locations, tissue thickness, correctly classified the tissue regions, and combines the information to recommend optimal suture locations. Limitations of this study include the small sample size and the use of flattened 2-D *ex vivo* tissue.^33^ Future research should focus on acquiring more *ex vivo* tissue data to allow separation of training and test datasets and to compare to ground truth. Another important step is to translate these findings to *in vivo* studies on tissues. Other tissue characteristics such as tissue perfusion, which is important for healing and can be detected using MSI,^34^ should also be included in the analysis and suture location optimization. Such suture maps processed in real time may potentially provide access to the best tissue information for anastomosis and thus mitigate the highly variable experiences and intraoperative decisions of surgeons. These suture maps showing the optimal suturing regions could also provide guidance to automated surgical procedures, where robots assist surgeons^35^ in performing safer operations with higher precision in less time.

## 5 Conclusion

To the best of our knowledge, this study demonstrates for the first time the feasibility of an MSI platform for the identification of blood vessels, differentiation between thin and thick tissue areas, and segmentation of different tissue types. The information is useful in determination of the optimal suture placements, which contributes to the development of a safer operation with reduced complications.
